# High-density lipoprotein cholesterol to low-density lipoprotein cholesterol ratio in early assessment of disease severity and outcome in patients with acute pancreatitis admitted to the ICU

**DOI:** 10.1186/s12876-020-01315-x

**Published:** 2020-05-27

**Authors:** Qin Wu, Xi Zhong, Min Fu, Hao Yang, Hong Bo, Xuelian Liao, Zhi Hu, Bo Wang, Zhongwei Zhang, Xiaodong Jin, Yan Kang

**Affiliations:** Department of Critical Care Medicine, West China Hospital, Sichuan University, Chengdu, China

**Keywords:** Acute pancreatitis, High-density lipoprotein cholesterol, low-density lipoprotein cholesterol, Mortality

## Abstract

**Background:**

Patients with acute pancreatitis usually exhibit dyslipidemia and oxidative stress. However, the significance of high-density lipoprotein cholesterol (HDL-C) level, low-density lipoprotein cholesterol (LDL-C) level and the HDL-C/LDL-C ratio (H/L ratio) as markers for disease progression remain unknown.

**Aim:**

The aim of this study was to evaluate the role of HDL-C levels, LDL-C levels and the H/L ratio as markers of disease progression in patients admitted to the intensive cate unit with acute pancreatitis.

**Methods:**

This retrospective study was conducted at a tertiary critical care center in China. Plasma HDL-C and LDL-C levels were measured in 166 patients with acute pancreatitis. The associations between HDL-C, LDL-C, H/L ratio, as well as other inflammatory index and mortality, were analyzed. Multivariate cox analysis based on two models was used to determine the independent prognostic factor. Predictive ability of in-hospital mortality for variables was determined using the receiver operating characteristics curves.

**Results:**

Significantly higher H/L ratios at admission were observed in patients with acute pancreatitis who died compared with survivors (0.93 vs. 0.64, *p* < 0.001). The area under the ROC curve for H/L ratio–based prediction of mortality was 0.658. When clinical confounders were included in multivariable cox regression analysis, the association was preserved (Model A HR = 1.587, *p* = 0.011; Model B HR = 1.332, *p* = 0.032). The mortality risk in different groups defined by an H/L ratio cutoff value was significantly different, based on survival curve analysis.

**Conclusion:**

The H/L ratio at the time of admission to the ICU appears to be a biomarker of disease progression in patients with acute pancreatitis.

## Background

Acute pancreatitis (AP), an inflammatory disease of the pancreas, is the leading cause of admission to hospital for gastrointestinal disorders in China and many other countries [1, 2]. Characterized by local and systemic inflammation, the severity of AP can range from mild to severe [3]. Although most patients with AP have the mild form of the disease, patients with moderate to severe AP, especially those who are admitted to the intensive care unit (ICU) due to organ failure, constitute a substantial burden to the Chinese medical system [4, 5]. Early assessment of disease severity and prognosis is crucial in defining treatment strategies, because effective treatment can significantly reduce mortality in patients with AP [6].

Systemic inflammation in patients with AP induces the release of free oxygen radicals through an immune-mediated mechanism that has been suggested to contribute to the pathogenesis of inflammatory diseases, and is believed to be associated with lipid profile changes due to impaired pancreatic exocrine secretion and endocrine function [7]. Recently, the relationship between low-density lipoprotein cholesterol (LDL-C), high-density lipoprotein cholesterol (HDL-C), cholesterol levels and pancreatic function has garnered much attention [8]. The HDL-C/LDL-C ratio (H/L ratio) is a powerful risk predictor and has been used as a biomarker not only for cardiovascular disease but also for ankylosing spondylitis and other diseases [9, 10]. However, the relationship between HDL-C and LDL-C levels, H/L ratio and outcome in patients with AP has not been confirmed. In this study, we aimed to investigate the association of LDL-C levels, HDL-C levels and H/L ratio with severity and outcome in patients with AP, comparing their predictive value with the traditional predictive values.

## Methods

### Study population

This was a retrospective database cohort study that was conducted in Department of Critical Care Medicine at West China Hospital in China. The study was approved by the institutional review board of West China Hospital. Informed consent from individuals was waived due to the retrospective, observational and anonymous nature of the study. The study included consecutive patients admitted to the Department of Critical Care Medicine at West China Hospital between January 2016 and December 2017. We prospectively established a patient database for our study, and information was collected automatically by computers or manually by attending physicians. The integrity of the data was monitored using a specialized system (Clinical Information System for Intensive Care Practitioners, Sichuan Smart Medical Information Systems, Chengdu, China).

### Study protocol

First, we scanned medical records from all patients discharged from ICU during the study period for a diagnosis of AP. The AP was diagnosed based on a combination of symptoms, physical examination and key laboratory values. Two of the following three characteristics were required: 1) upper abdominal pain during acute episodes, 2) serum amylase or lipase levels three times above normal levels, and 3) abdominal imaging findings consistent with AP. The exclusion criteria were as follows: a recurrence of chronic pancreatitis, the inability to obtain laboratory measurements or medical records, hospitalization more than 48 h after onset of abdominal pain, age under 18, and stay in the ICU of less than 24 h. For patients with more than one stay in the ICU during the study period, the first episode was retained for further analysis. Three levels of severity were defined according to the revised Atlanta classification of AP. For the etiological diagnosis, gallstone-related AP can be diagnosed if the images (computed tomography, magnetic resonance imaging or ultrasonography) show the definitive presence of gallstones. Hyperlipidemic pancreatitis is defined as severe serum hyperlipidemia when triglyceride levels are greater than 11.3 mmol/L, or in excess of 5.65 mmol/L in patients with grossly lipemic serum. A patient would be diagnosed as having alcoholic pancreatitis if the patient had a history of alcohol use before the AP episode and consumed more than 50 g/day for more than 5 years [11].

### Data collection

The data that are recorded routinely in our hospital at admission include the severity of the disease. The Acute Physiology and Chronic Health Evaluation II (APACHE II) score and Ranson’s score were calculated by the attending physician who saw the patient at admission. Cholesterol, HDL-C and LDL-C levels were evaluated by laboratory tests after admission and whenever required according to the judgment of the attending physician (available 24 h/day). Arterial was collected for air blood gas analysis whenever necessary. Usually, laboratory tests are performed within 4 h of admission and evaluated at least once per day during an ICU stay. Laboratory values were measured within 2 h after blood collection in the Department of Laboratory Medicine at West China Hospital. Cholesterol, HDL-C and LDL-C levels were measured directly using a Biochemical Analyzer (Dimension AR/AVL Clinical Chemistry System, Newark, NJ, USA). Other laboratory parameters obtained as part of our study included red blood cell count (RBC), hemoglobin (HGB), hematocrit (HCT), platelet count (PLT), white blood cell count (WBC); activated partial thromboplastin time (APTT), fibrinogen (Fib), thrombin time (TT), arterial oxygen partial pressure (PaO2), arterial carbon dioxide partial pressure (PaCO2), lactate (Lac), procalcitonin (PCT),c-reactive protein (CRP), interleukin 6 (IL-6), total bilirubin (TB), direct bilirubin (DB), alanine aminotransferase (ALT), aspartate aminotransferase (AST), creatinine (Crea), albumin (ALB), glucose (Glu), cystatin C (Cys-c), gamma-glutamyl transferase (GGT), LDL-C, HDL-C and TG.

### Statistical analyses

We analyzed our data using SPSS Statistics 25 (IBM Corp., Armonk, NY, USA) and GraphPad Prism (GraphPad Software, San Diego, CA, USA), with significance defined as *P* < 0.05. Continuous variables were presented as means ± standard deviation (SD) or median and interquartile range (IQR) and tested for distribution normality using the Kolmogorov-Smirnov test. Categorical variables are expressed as percentages. The Student’s t-test or Mann-Whitney U was used for quantitative data based on normality assumption. Categorical variables were analyzed in using the Fisher exact test. Pearson’s correlation analysis was used to assess the correlation between variables. A multivariable cox regression analysis was performed to examine the association of variables with the risk of death after adjustment for potential confounders (Model A for age, gender and etiology, underlying medical conditions and severity; Model B for age, gender and etiology, underlying medical conditions, severity, APACHE II score and Ranson’s score). Results of regression analysis were demonstrated as hazards ratio with corresponding 95% confidence interval (CI). Receiver operating characteristics (ROC) curves were constructed and the area under the curve (AUC) as well as the corresponding 95% CI was assessed as a summary measure of predictive accuracy of variables for mortality. Log-rank tests were used to assess the statistical difference between the survival curves constructed using the Kaplan-Meier method.

## Results

**Fig. 1:**
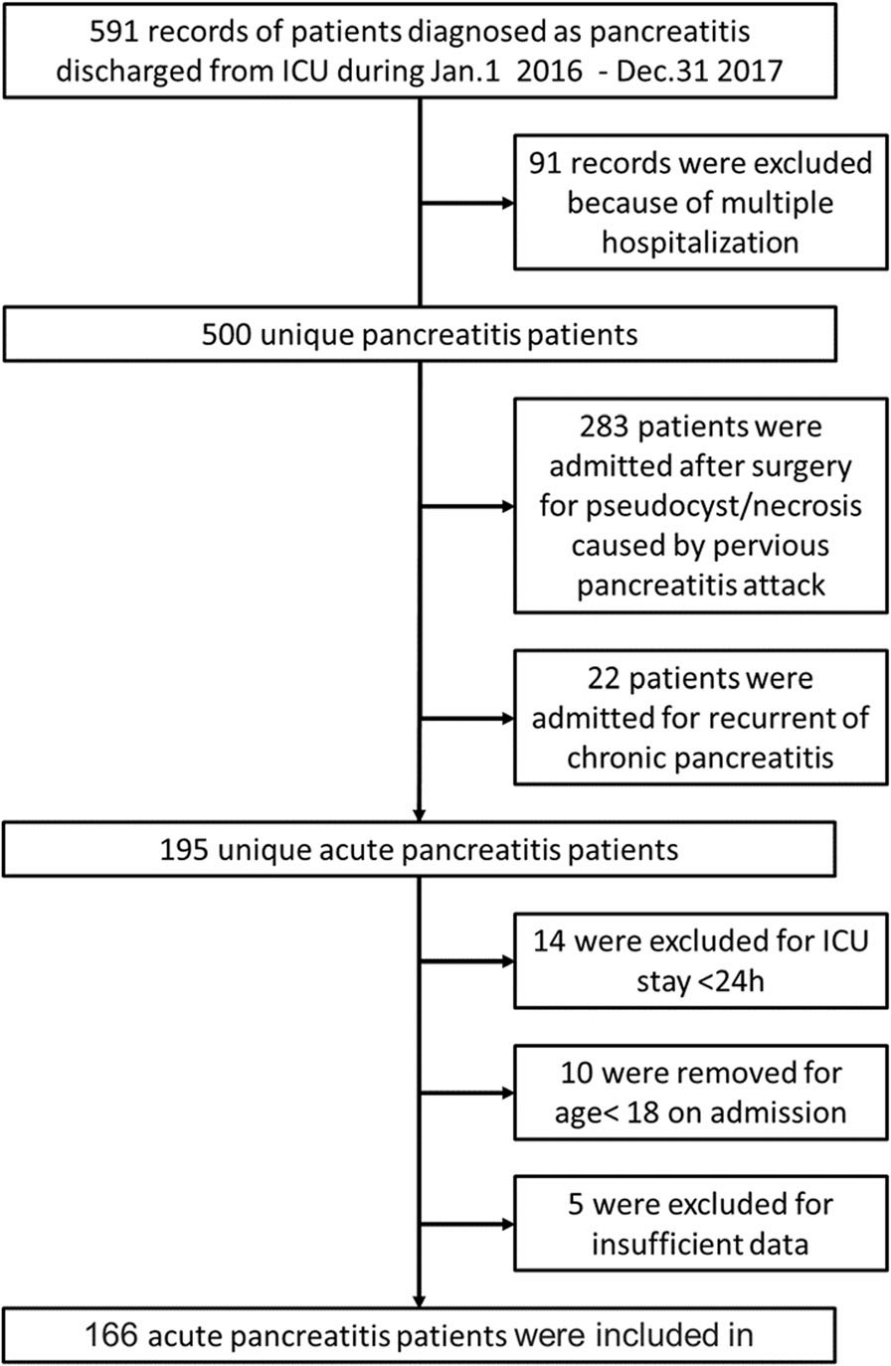
Flowchart of current study. A total of 500 unique acute pancreatitis patients was retrospectively collected. Ten were excluded for the reason of age less than 18 years when admission, 14 for ICU stay less than 24 h, 5 for insufficient data and 166 acute pancreatitis patients were finally enrolled in this study for further analysis

A total of 591 records from patients diagnosed with pancreatitis and discharged from the ICU during our study period were initially included. For patients who had more than one ICU stay during the study period, the medical records from the first stay only were retained, resulting in the elimination of 91 records from re-admitted patients. Of the 500 unique patients, 22 were hospitalized for recurrence of chronic pancreatitis and 283 were admitted to hospital for postoperative necrosis or pseudocysts caused by previous episodes of AP. In addition, 10 patients were younger than 18 years of age at admission. Fourteen patients were in the ICU for less than 24 h, and five were excluded due to insufficient data. Ultimately, the records for 166 patients with AP were retained for further analysis (Fig. 1). The baseline characteristics of the study population are summarized in Table 1 and Supplementary Table 1. The average age of the entire cohort was 47.49 ± 15.06 years, and there were more men than women. Gallstones were the most common cause of AP in these patients. The average APACHE II score and Ranson’s score were 19.27 ± 7.32 and 4.24 ± 1.55, respectively. Severe AP was the most common type of AP observed in this cohort. The total in-hospital mortality rate was 27.11%, and the mean ICU stay was 23.37 ± 22.40 days.

**Table 1:** Demographics, Clinical and Outcome data of acute pancreatitis patient cohort

Parameters	All (***n*** = 166)	Non-Survivor (***n*** = 45)	Survivor (***n*** = 121)	P
Demographic Data				
Age, mean (SD),y	47.49 (15.06)	49.67 (13.73)	46.68 (15.53)	0.310
Male, n (%)	97 (58.43)	25 (55.60)	72 (59.50)	0.646
Etiology, n (%)				0.007
Biliary	71 (42.80)	15 (33.30)	56 (46.30)	
Hyperlipidemia	34 (20.50)	5 (11.10)	29 (24.00)	
Others	61 (36.70)	25 (55.60)	36 (29.80)	
Underlying medical conditions				
Hypertension, n (%)	24 (14.50)	5 (11.10)	19 (15.70)	0.455
Diabetes Mellitus, n (%)	28 (16.90)	5 (11.10)	23 (19.00)	0.227
Severity, n (%)				0.533
Moderately severe	27 (16.30)	6 (13.30)	21 (17.40)	
Severe	139 (83.70)	39 (86.70)	100 (82.60)	
APACHEIIScore^a^, mean (SD)	19.27 (7.32)	22.53 (5.69)	18.03 (7.50)	< 0.001
Ranson’s Score^a^, mean (SD)	4.24 (1.55)	4.29 (1.61)	4.11 (1.36)	0.493
CHOL^a^, mean (SD), mmol/L	3.41 (2.79)	3.00 (3.42)	3.00 (3.42)	0.257
HDL-C^a^, mean (SD), mmol/L	0.39 (0.28)	0.26 (0.13)	0.44 (0.31)	< 0.001
LDL-C^a^, mean (SD), mmol/L	0.91 (0.70)	0.54 (0.53)	1.05 (0.71)	< 0.001
H/L ratio^a^, mean (SD)	0.72 (0.92)	0.93 (1.29)	0.64 (0.72)	< 0.001
Statins usage**, n (%)	2 (1.20)	1 (2.22)	1 (0.82)	0.464
Ventilation free days^b^, mean (SD), d	16.87 (8.93)	13.64 (9.16)	18.07 (8.56)	0.004
Renal replacement therapy, n (%)	27 (16.30)	12 (26.70)	15 (12.40)	0.149
Hospital cost, mean (SD), CHY	182,392.76 (141,028)	229,767 (163.568)	164,627 (127,869)	0.008
ICU LOS, mean (SD), d	23.37 (22.40)	20.91 (16.56)	24.28 (24.22)	0.391
Hospital LOS, mean (SD), d	30.54 (23.30)	23.27 (17.18)	33.27 (24.73)	0.014

*APACHE* Acute Physiology and Chronic Health Evaluation, *CHOL* cholesterol, *HDL-C* High-density lipoprotein cholesterol, *LDL-C* Low-density lipoprotein cholesterol, *H/L* HDL-C/ LDL-C, *LOS* length of stay

^a^on admission

^b^within 28 days

**Fig. 2:**
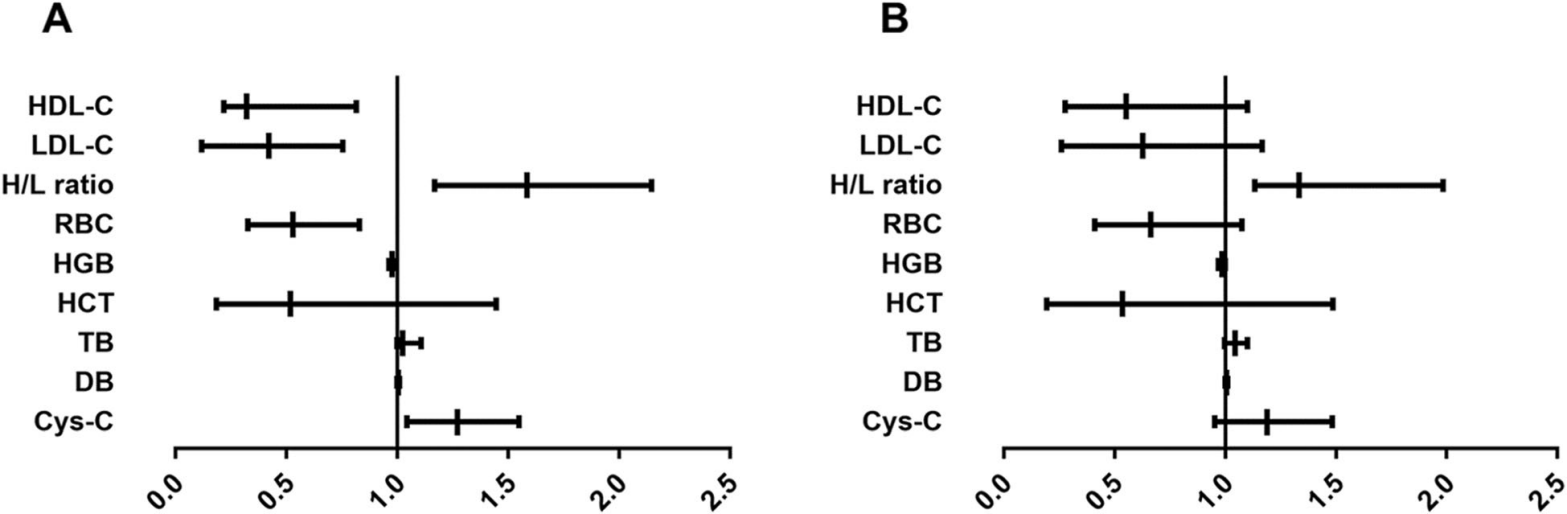
Forest plots from multivariate Cox regression models: **a** adjust for age, gender and etiology, underlying medical conditions and severity and (**b**) adjust for age, gender and etiology, underlying medical conditions, severity, APACHE II score and Ranson’s score. RBC: Red blood cell, HGB: Hemoglobin, HCT: Hematocrit, TB: Total bilirubin, DB: Direct bilirubin, Cys-c: Cystatin C, HDL-C: High-density lipoprotein cholesterol, LDL-C: Low-density lipoprotein cholesterol, H/L: HDL-C/ LDL-C

We divided our AP patients into two groups based on whether or not they survived the hospital stay. There was no statistical difference in the age or gender of survivors and non-survivors (Table 1). However, a significant difference was observed between these patients in terms of etiology (*p* = 0.007), APACHE II score (*p* < 0.001), HDL-C level (*p* < 0.001), LDL-C level (*p* < 0.001), H/L ratio (*p* < 0.001), RBC (*p* = 0.001), HGB (*p* = 0.001), HCT (*p* = 0.001), TB (*p* = 0.003), DB (*p* = 0.002) and Cys-C (*p* < 0.001). Multivariate Cox analysis based on two models showed that the H/L ratio was an independent prognostic factor for patients with AP (Table 2, Fig. 2). Moreover, in patients with gallstone AP, the H/L ratio was significantly different between groups (Supplementary Material 2). In addition, there was a significant correlation between the H/L ratio and the APACHE II score (*p* < 0.001, r = 0.445) and Ranson’s score (*p* = 0.018, r = 0.229). In addition, the trend analysis shown in Table 3 indicated that an increased H/L ratio was associated with increased in-hospital mortality.

**Table 2:** Multivariate Cox analysis for HDL-C, LDL-C and H/L ratio associated with in-hospital mortality

variables	Hazard Ratio	95% CI		***P*** value
		lower	upper	
Model A				
HDL-C	0.322	0.218	0.815	0.010
LDL-C	0.421	0.119	0.754	0.024
H/L ratio	1.587	1.169	2.146	0.011
Model B				
HDL-C	0.553	0.279	1.099	0.091
LDL-C	0.628	0.262	1.168	0.132
H/L ratio	1.332	1.133	1.985	0.032

Model A adjust for age, gender and etiology, underlying medical conditions and severity;

Model B adjust for age, gender and etiology, underlying medical conditions, severity, APACHE II score and Ranson’s score

*HDL-C* High-density lipoprotein cholesterol, *LDL-C* Low-density lipoprotein cholesterol, *H/L* HDL-C/ LDL-C

95% CI: 95% confidence interval

**Table 3:** Outcomes of patients stratifying based on H/L ratio quartile

Parameters	Patient number	In hospital death	Adjusted Hazard Ratio (95% CI)		
			Model 1^a^	Model 2^**b**^	Model 3^**c**^
Quartile 1	41	6	1.00 (reference)	1.00 (reference)	1.00 (reference)
Quartile 2	42	9	1.573 (1.190–1.848)	1.539 (0.985–1.815)	0.784 (0.245–2.559)
Quartile 3	41	14	2.844 (1.691–4.920)	1.777 (1.045–4.774)	1.796 (0.681–5.731)
Quartile 4	42	16	4.188 (1.832–5.754)	2.144 (1.804–5.720)	2.077 (0.753–5.733)
P for trend			0.006	0.022	0.039

^a^ Crude, no adjustment

^b^Adjusted for age, gender, etiology, underlying medical conditions, and severity

^c^ Adjusted for age, gender, etiology, underlying medical conditions, severity, APACHE II score and Ranson’s score

**Fig. 3:**
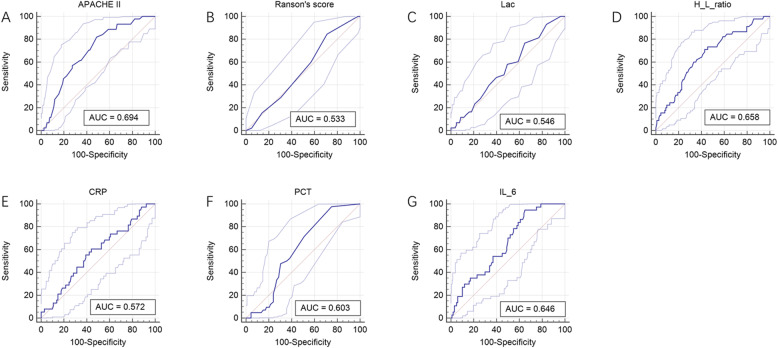
Receiver operating characteristic (ROC) curves of the APACHE II score, Ranson’s score, lactate, H/L ratio, C-reactive protein, procalcitonin, and interleukin 6 level in predicting mortality after ICU admission in acute pancreatitis patients

**Fig. 4:**
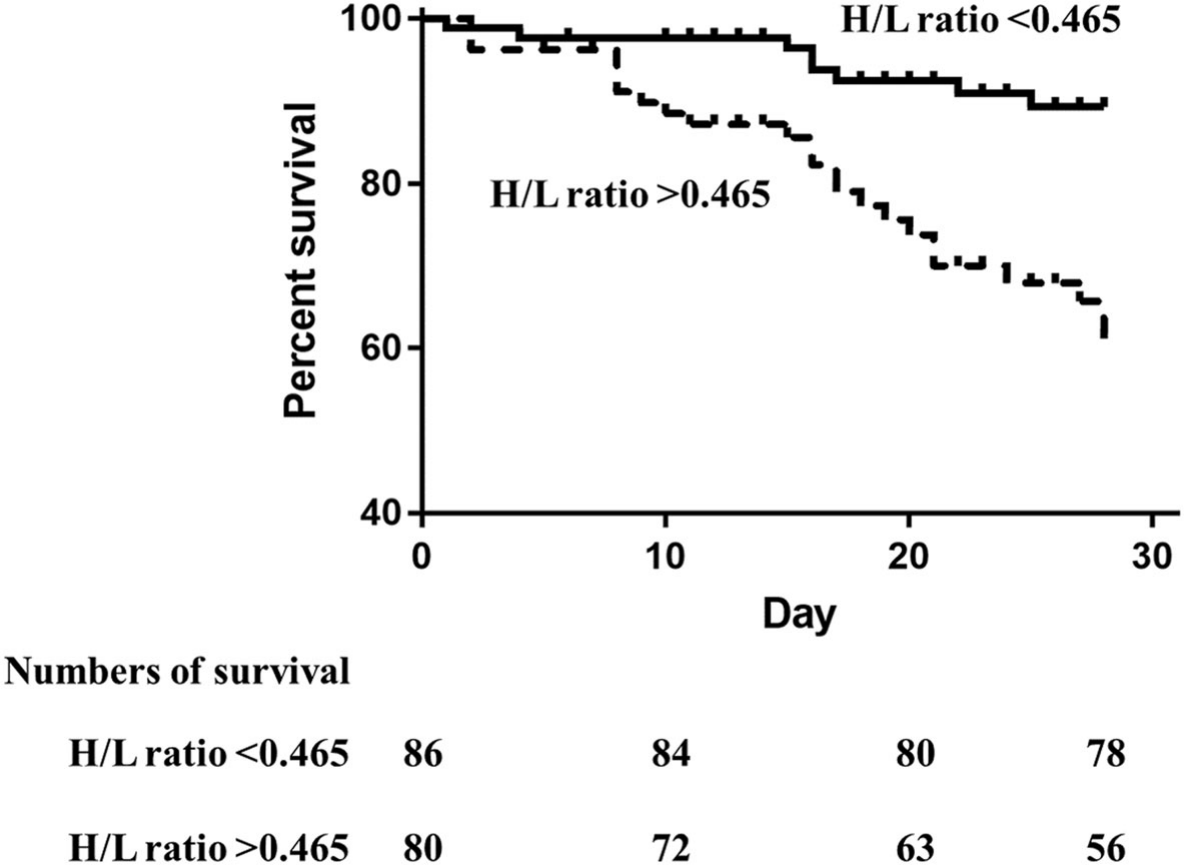
Survival analysis between groups based on an H/L ratio level cutoff. Kaplan–Meier survival curves were created based on H/L ratio cutoff values of 0.465. A significant difference was observed between the two curves

We then compared the diagnostic performances of APACHE II score, Ranson’s score, Lac, PCT, CRP, IL-6 and H/L ratio using the AUC for discrimination mortality. The AUCs for APACHE II score, Ranson’s score, Lac, PCT, CRP, IL-6 and H/L ratio were 0.694 ([95% CI] 0.604–0.781), 0.533 ([95% CI] 0.458–0.619), 0.546 ([95% CI] 0.469–0.659), 0.603 ([95% CI] 0.529–0.715), 0.572 ([95% CI] 0.484–0.673), 0.646 ([95% CI] 0.563–0.745) and 0.658 ([95% CI] 0.564–0.747), respectively (Fig. 3). Kaplan-Meier survival curves were established based on calculating the minimum d (H/L ratio, 0.465) using cutoff values determined from the receiver operating characteristic curves. After stratification according to H/L ratio, the 28-day survival rates for patients with AP were significantly different (Fig. 4). Patients with a lower H/L ratio at admission had a higher survival rate than patients with a higher H/L ratio (log-rank test, *p* < 0.001, Table 4). In addition, using this threshold (H/L ratio, 0.465), the sensitivity of the H/L ratio in discriminating between non-survivors and survivors was 71.10%, with a specificity of 57.0%.

**Table 4:** Clinical data of the acute pancreatitis patient cohort based on H/L ratio

Parameters	H/L ratio < 0.465 (***n*** = 86)	H/L ratio > 0.465 (***n*** = 80)	P
Demographic Data			
Age, mean (SD),y	45.71 (13.67)	49.18 (16.20)	0.141
Male, n (%)	45 (52.33)	52 (65.00)	0.098
Etiology, n (%)			0.031
Biliary	36 (41.86)	35 (43.80)	
Hyperlipidemia	24 (27.91)	10 (12.50)	
Others	26 (30.23)	35 (43.80)	
Underlying medical conditions			
Hypertension, n (%)	6 (6.97)	18 (22.50)	0.004
Diabetes Mellitus, n (%)	14 (16.28)	14 (17.50)	0.834
Severity, n (%)			0.034
Moderately severe	19 (22.09)	8 (10.00)	
Severe	67 (77.91)	72 (90.00)	
APACHEIIScore*, mean (SD)	17.99 (6.50)	20.50 (7.85)	0.027
Ranson’s Score*, mean (SD)	4.18 (1.46)	4.30 (1.64)	0.635
CHOL, mean (SD), mmol/L	3.63 (2.61)	3.20 (2.96)	0.323
HDL-C, mean (SD), mmol/L	0.33 (0.17)	0.44 (0.36)	0.015
LDL-C, mean (SD), mmol/L	1.25 (0.72)	0.59 (0.51)	< 0.001
H/L ratio, mean (SD)	0.28 (0.09)	1.14 (1.15)	< 0.001
Ventilation free days#, mean (SD), d	16.13 (9.16)	17.57 (8.71)	0.045
Renal replacement therapy, n (%)	6 (6.97)	21 (26.25)	0.001
ICU LOS, mean (SD), d	25.86 (26.62)	21.02 (17.43)	0.163
Hospital LOS, mean (SD), d	33.40 (22.88)	27.85 (23.50)	0.126
Complete blood count*			
RBC, mean (SD),× 10^9^/L	3.15 (0.69)	3.21 (1.00)	0.649
HGB, mean (SD), g/L	95.34 (22.37)	95.12 (27.54)	0.955
HCT, mean (SD), L/L	0.29 (0.06)	0.29 (0.08)	0.844
PLT, mean (SD),×10^9^/L	156.36 (104.59)	144.06 (111.11)	0.463
WBC, mean (SD),×10^9^/L	11.95 (5.18)	12.74 (6.52)	0.389
Coagulation test*			
APTT, mean (SD), s	36.57 (10.40)	44.76 (19.51)	0.001
Fib, mean (SD), g/L	5.07 (1.90)	4.13 (1.77)	0.001
TT, mean (SD), s	19.52 (12.51)	18.05 (2.65)	0.290
Arterial Blood Gas Test*			
pH, mean (SD)	7.36 (0.27)	7.28 (0.46)	0.193
PaO2, mean (SD), mmHg	84.18 (29.66)	95.73 (41.32)	0.045
PaCO2, mean (SD), mmHg	40.25 (6.77)	39.50 (9.84)	0.573
Lac, mean (SD), mmol/L	1.63 (0.49)	1.96 (1.02)	0.012
Biochemical analysis*			
PCT, mean (SD), ng/mL	6.82 (16.18)	10.44 (17.42)	0.184
CRP, mean (SD), mg/L	189.16 (105.01)	183.75 (92.43)	0.747
IL-6, mean (SD), ng/mL	287.52 (481.42)	459.90 (752.33)	0.114
Serum sodium, mean (SD), mmol/L	137.66 (6.60)	140.05 (7.72)	0.035
Serum potassium, mean (SD), mmol/L	3.75 (0.41)	3.81 (0.50)	0.368
Serum chloride, mean (SD), mmol/L	108.05 (6.33)	109.45 (6.95)	0.181
TB, mean (SD), mmol/L	40.29 (57.13)	41.17 (53.33)	0.918
DB, mean (SD), mmol/L	30.97 (47.86)	33.20 (46.99)	0.763
ALT, mean (SD), mmol/L	50.06 (167.93)	92.73 (199.87)	0.140
AST, mean (SD), mmol/L	78.55 (228.91)	164.60 (465.49)	0.137
ALB, mean (SD), mmol/L	30.74 (5.11)	29.41 (6.21)	0.135
Glu, mean (SD), mmol/L	10.07 (3.11)	10.06 (3.72)	0.982
Crea, mean (SD), mmol/L	141.64 (140.04)	212.41 (196.63)	0.009
Cys-c, mean (SD), mmol/L	1.43 (0.83)	1.93 (1.35)	0.005
GGT, mean (SD), mmol/L	108.49 (123.97)	91.62 (108.70)	0.352
TG, mean (SD), mmol/L	3.56 (2.26)	4.07 (5.21)	0.419

*APACHE* Acute Physiology and Chronic Health Evaluation, *RBC* Red blood cell, *HGB* Hemoglobin, *HCT* Hematocrit, *MCV* Mean corpuscular volume, *MCH* Mean corpuscular hemoglobin, *MCHC* Mean corpuscular hemoglobin concentration, *RDW* Red blood cell distribution width, *PLT* Platelets, *WBC* white blood cell, *INR* International normalized ratio, *PT* prothrombin time, *APTT* Activated partial thromboplastin time, *Fib* fibrinogen, *TT* Thrombin time, *PaO2* Arterial oxygen partial pressure, *PaCO2* Arterial carbon dioxide partial pressure, *BE* Base excess, *Lac* lactate, *PCT* Procalcitonin, *TB* Total bilirubin, *DB* Direct bilirubin, *ALT* Alanine Aminotransferase, *AST* Aspartate Aminotransferase, *ALP* Alkaline Phosphatase, *TP* Total protein, *ALB* Albumin, *Glu* Glucose, *Crea* Creatinine, *Cys-c* Cystatin C, *GGT* Gamma-Glutamyl Transferase, *TG* Triglycerides, *CHOL* cholesterol, *HDL-C* High-density lipoprotein cholesterol, *LDL-C* Low-density lipoprotein cholesterol, *H/L* HDL-C/ LDL-C, *LOS* length of stay, *IQR* interquartile range

## Discussion

In this study, we evaluated the relationship between lipid levels at the time of admission and hospital mortality in patients with AP. We found that higher H/L ratio levels were positively correlated with increased mortality in patients with AP, and determined the optimal cutoff for using the H/L ratio to assess AP-related mortality. The main results from this study indicate that the H/L ratio can be used to predict outcomes in patients with AP. To the best of our knowledge, this is the first study to confirm the predictive value of the H/L ratio and analyze the accuracy of this indicator in patients with AP.

Cholesterol is a structural component of cell membranes and is localized in membrane micro-domains that assemble signal transduction machinery. It is associated with proteins involved in key cell signaling pathways that are closely related to inflammation [8, 12]. There are five major lipoproteins: chylomicrons and very low-density lipoprotein, medium-density lipoprotein, low-density lipoprotein and high-density lipoprotein, which are classified compared with the density of the surrounding water [13, 14]. To facilitate cell absorption and utilization of fat through receptor-mediated endocytosis, LDL carries all of the fatty molecules, cholesterol, phospholipids and triglycerides, while HDL transports cholesterol from the cells and tissues back to the liver [15]. Rather than measuring actual HDL and LDL particles, assessing HDL-C and LDL-C levels provides information about how much cholesterol is delivered by all HDL and LDL particles. HDL and LDL oxidation is associated with an increased risk of developing cardiovascular disease because the oxidized forms are more susceptible to proteoglycan [16].

The relationship between HDL-C, LDL-C and cardiovascular disease has been well established over the years. Many guidelines recommend lowering LDL-C to reduce the risk of coronary heart disease, and highlight the protective role of HDL-C [17]. Given the essential function of blood lipids, LDL-C and HDL-C levels can be used to predict mortality not only in patients with cardiovascular disease but also in patients with colorectal cancer [18]. Although pancreatic exocrine function is directly linked to lipid metabolism, little is known about the predictive value of HDL-C and LDL-C in patients with AP.

In this retrospective study, we analyzed medical records from 166 patients with AP, who were admitted to the ICU for organ support, and evaluated the role of LDL-C and HDL-C levels and H/L ratios in predicting mortality in these patients. Interestingly, we found that the HDL-C/LDL-C ratio is a potential biomarker, and is more sensitive and specific than lactate, a biomarker that is widely recognized as a marker of mortality in critically ill patients [9].

The mechanism of the relationship between H/L ratios and AP prognosis is not clear, but there are several plausible explanations. Some studies have shown that there is some correlation between serum lipid levels and nutritional status, and the predictive value of H/L ratios could therefore be attributable to the nutritional status of patients with AP [9, 19]. However, there was no difference in serum albumin levels between survivors and non-survivors in our study. Therefore, it is unlikely that the different H/L ratios observed in these patients were related to nutritional status. Besides, we observe a inconsistent trend of cholesterol levels and triglyceride level in our cohort. Since cholesterol has an impact on maintaining vascular integrity and LDL-C levels can alter platelet acceptability through their effects on platelet activating factor, we hypothesize that the H/L ratio has a more complex role in AP progression, and believe that further research regarding this role is urgently needed.

AP may be caused by several factors. Gallstones and alcohol abuse are long-term risk factors, although hypertriglyceridemia is more common in China than alcohol abuse [20]. The method most commonly used to measure LDL-C is the Friedewald equation. Various confounding laboratory abnormalities can be present in patients with AP, especially in those with hypertriglyceridemia pancreatitis, and LDL-C levels are still the main target for treatment in patients with hyperlipidemia and the primary factor on which most of our clinical decisions are based [13]. In addition, in patients with simple gallstone AP, H/L ratios were still significantly different between survivors and non-survivors, suggesting that our conclusion is not related to AP etiology.

We compared the predicted values of common predictive indicators and the H/L ratio. In our study, H/L ratio had a higher predictive value than PCT, CRP or IL-6. Recently, several studies with small sample sizes reported that low levels of HDL-C are associated with high risk of persistent organ failure in AP [21]. Our study confirmed that HDL-C levels could be a predictor for patients with AP, and we found that the H/L ratio had a better predictive value than HDL-C levels. However, we found lower HDL-C is beneficial for patients, which is consistent with other studies [22, 23]. One explanation is that due to the retrospective study design, the lipid status parameters in our study are influenced by some patients leading to the distribution of our data is unlikely with others. In the future we still need more well-designed research to clarify relationships between HDL-C and AP mortality.

Our study had several limitations. First, because of the small sample size and the retrospective evaluation of cases, selection bias may have affected the generalizability of our results. Second, since this was an observational single-center study, the causal role of LDL-C in AP requires further investigation in prospective multicenter validation studies. In addition, we only examined one-time measurements. Therefore, this study did not address the issue of HDL-C and HDL-C variation in individuals. We use morality as our primary outcome instead of organ failure assessment which is not available for all patients because of the study design though it is not an indicator early enough for AP patients’ stratification in the ICU. There is an urgent need to obtain additional prospective data in consecutive patients to confirm our findings.

## Conclusions

In summary, our results suggest that higher H/L ratios are independently associated with poor progression in patients with AP who are admitted to the ICU. Although our study is preliminary, we speculate that serum H/L ratios may be a predictive biomarker for mortality in patients with AP.

## Supplementary information

**Additional file 1: Supplementary Data 1.** Clinical data of acute pancreatitis patient cohort.

**Additional file 2: Supplementary Table 2.** Demographics, Clinical and Outcome data of gallstone acute pancreatitis patient cohort.

## Data Availability

The datasets generated and analyzed in this article are not publicly available due to health privacy concerns. However, they are available from the corresponding author and will be obtainable by the public when the database construction is complete.

## Abbreviations

HDL-C: High-density lipoprotein cholesterol
LDL-C: Low-density lipoprotein cholesterol
H/L: HDL-C/LDL-C
AP: Acute pancreatitis
ICU: Intensive care unit
APACHE II: The Acute Physiology and Chronic Health Evaluation II
RBC: Red blood cell count
HGB: Hemoglobin
HCT: Hematocrit
PLT: Platelet count
WBC: White blood cell count
APTT: Activated partial thromboplastin time
Fib: Fibrinogen
TT: Thrombin time
PaO2: Arterial oxygen partial pressure
PaCO2: Arterial carbon dioxide partial pressure
Lac: Lactate
PCT: Procalcitonin
CRP: C-reactive protein
IL-6: Interleukin 6
TB: Total bilirubin
DB: Direct bilirubin
ALT: Alanine aminotransferase
AST: Aspartate aminotransferase
Crea: Creatinine
ALB: Albumin
Glu: Glucose
Cys-c: Cystatin C
GGT: Gamma-glutamyl transferase
SD: Standard deviation
IQR: Interquartile range
AUC: Area under the receiver
