# Supplementary material for: High-density lipoprotein cholesterol to low-density lipoprotein cholesterol ratio in early assessment of disease severity and outcome in patients with acute pancreatitis admitted to the ICU
Source: BMC Gastroenterol. 2020 May 27;20:164. doi: 10.1186/s12876-020-01315-x (PMC7254649; doi:10.1186/s12876-020-01315-x)
Supplement: Supplementary file 1 — Additional file 1: Supplementary Data 1. Clinical data of acute pancreatitis patient cohort. [file 12876_2020_1315_MOESM1_ESM.docx]

**Supplementary data 1. Clinical data of acute pancreatitis patient cohort**

| **Parameters** | **All (n=166)** | **Non-Survivor (n=45)** | **Survivor (n=121)** | **P** |
| --- | --- | --- | --- | --- |
| Complete blood count* |  |  |  |  |
| RBC, mean (SD),×10^9^/L | 3.18 (0.86) | 2.83 (0.73) | 3.31 (0.87) | 0.001 |
| HGB, mean (SD), g/L | 95.22 (25.11) | 85.09 (21.75) | 98.99 (25.31) | 0.001 |
| HCT, mean (SD), L/L | 0.29 (0.07) | 0.26 (0.06) | 0.30 (0.07) | 0.001 |
| PLT, mean (SD),×10^9^/L | 149.99 (107.86) | 160.36 (116.21) | 146.13 (104.84) | 0.452 |
| WBC, mean (SD),×10^9^/L | 12.36 (5.91) | 12.49 (6.29) | 12.31 (5.78) | 0.861 |
| Coagulation test* |  |  |  |  |
| APTT, mean (SD), s | 41.81 (16.26) | 44.18 (17.26) | 39.55 (15.77) | 0.103 |
| Fib, mean (SD), g/L | 4.58 (1.86) | 4.61 (1.69) | 4.57 (1.92) | 0.924 |
| TT, mean (SD), s | 18.76 (8.89) | 17.64 (1.84) | 19.18 (10.36) | 0.324 |
| Arterial Blood Gas Test* |  |  |  |  |
| pH, mean (SD) | 7.32 (0.39) | 7.30 (0.38) | 7.33 (0.38) | 0.707 |
| PaO2, mean (SD), mmHg | 90.20 (36.56) | 92.27 (40.48) | 89.40 (35.08) | 0.657 |
| PaCO2, mean (SD), mmHg | 39.86 (8.47) | 38.67 (10.71) | 40.31 (7.47) | 0.270 |
| Lac, mean (SD), mmol/L | 1.80 (0.82) | 1.93 (1.14) | 1.75 (0.64) | 0.229 |
| Biochemical analysis* |  |  |  |  |
| PCT, mean (SD), ng/mL | 8.68 (16.87) | 6.61 (9.54) | 9.45 (18.22) | 0.353 |
| CRP, mean (SD), mg/L | 186.38 (98.42) | 204.70 (99.68) | 179.56 (97.55) | 0.108 |
| IL-6, mean (SD), ng/mL | 375.60 (638.05) | 528.86 (741.03) | 318.89 (589.54) | 0.037 |
| Serum sodium, mean (SD), mmol/L | 138.91 (7.27) | 140.43 (7.61) | 138.34 (7.10) | 0.100 |
| Serum potassium, mean (SD), mmol/L | 3.78 (0.46) | 3.89 (0.55) | 3.74 (0.41) | 0.076 |
| Serum chloride, mean (SD), mmol/L | 108.78 (6.68) | 110.16 (7.13) | 108.26 (6.46) | 0.105 |
| TB, mean (SD), mmol/L | 40.75 (55.02) | 61.49 (83.74) | 33.03 (37.02) | 0.003 |
| DB, mean (SD), mmol/L | 32.13 (47.28) | 51.00 (71.32) | 25.11 (32.03) | 0.002 |
| ALT, mean (SD), mmol/L | 72.17 (185.84) | 68.84 (194.95) | 73.40 (183.16) | 0.899 |
| AST, mean (SD), mmol/L | 123.13 (372.25) | 149.22 (510.16) | 113.43 (307.82) | 0.583 |
| ALB, mean (SD), mmol/L | 30.05 (5.73) | 28.79 (5.29) | 30.52 (5.83) | 0.084 |
| Glu, mean (SD), mmol/L | 10.06 (3.43) | 10.70 (3.55) | 9.82 (3.38) | 0.150 |
| Crea, mean (SD), mmol/L | 178.30 (174.83) | 236.67 (193.71) | 156.60 (162.85) | 0.008 |
| Cys-c, mean (SD), mmol/L | 1.69 (1.16) | 2.30 (1.31) | 1.46 (1.01) | <0.001 |
| GGT, mean (SD), mmol/L | 99.75 (116.26) | 115.78 (138.21) | 93.79 (107.01) | 0.280 |
| TG, mean (SD), mmol/L | 3.82 (4.06) | 3.54 (3.01) | 3.93 (4.38) | 0.591 |

APACHE: Acute Physiology and Chronic Health Evaluation; RBC: Red blood cell, HGB: Hemoglobin, HCT: Hematocrit, MCV: Mean corpuscular volume, MCH: Mean corpuscular hemoglobin, MCHC: Mean corpuscular hemoglobin concentration, RDW: Red blood cell distribution width, PLT: Platelets, WBC: white blood cell; INR: International normalized ratio, PT: prothrombin time, APTT: Activated partial thromboplastin time, Fib: fibrinogen, TT: Thrombin time, PaO2: Arterial oxygen partial pressure, PaCO2: Arterial carbon dioxide partial pressure, BE: Base excess, Lac: lactate, PCT: Procalcitonin, TB: Total bilirubin, DB: Direct bilirubin, ALT: Alanine Aminotransferase, AST: Aspartate Aminotransferase, ALP: Alkaline Phosphatase, TP: Total protein, ALB: Albumin, Glu: Glucose, Crea: Creatinine, Cys-c: Cystatin C, GGT: Gamma-Glutamyl Transferase, TG: Triglycerides, CHOL: cholesterol, HDL-C: High-density lipoprotein cholesterol, LDL-C: Low-density lipoprotein cholesterol, H/L: HDL-C/ LDL-C, LOS: length of stay; IQR: interquartile range

*on admission

^#^total amount during ICU stay
