# Supplementary material for: High-density lipoprotein cholesterol to low-density lipoprotein cholesterol ratio in early assessment of disease severity and outcome in patients with acute pancreatitis admitted to the ICU
Source: BMC Gastroenterol. 2020 May 27;20:164. doi: 10.1186/s12876-020-01315-x (PMC7254649; doi:10.1186/s12876-020-01315-x)
Supplement: Supplementary file 2 — Additional file 2: Supplementary Table 2. Demographics, Clinical and Outcome data of gallstone acute pancreatitis patient cohort. [file 12876_2020_1315_MOESM2_ESM.docx]

**Supplementary table 2. Demographics, Clinical and Outcome data of gallstone acute pancreatitis patient cohort**

| **Parameters** | **All (n=71)** | **Non-Survivor (n=15)** | **Survivor (n=56)** | **P** |
| --- | --- | --- | --- | --- |
| Demographic Data |  |  |  |  |
| Age, mean (SD),y | 51.17 (16.07) | 56.47 (11.84) | 49.75 (16.80) | 0.151 |
| Male, n (%) | 38 (53.52) | 7 (46.70) | 31 (55.40) | 0.549 |
| Underlying medical conditions |  |  |  |  |
| Hypertension, n (%) | 15 (21.13) | 2 (13.30) | 13 (23.20) | 0.405 |
| Diabetes Mellitus, n (%) | 14 (19.72) | 1 (6.70) | 13 (23.20) | 0.153 |
| Severity, n (%) |  |  |  | 0.683 |
| Moderately severe | 13 (18.31) | 2 (13.30) | 11 (19.60) |  |
| Severe | 58 (81.69) | 13 (86.70) | 45 (80.40) |  |
| APACHEⅡScore*, mean (SD) | 18.75 (6.55) | 21.70 (5.41) | 17.94 (6.65) | 0.032 |
| Ranson’s Score*, mean (SD) | 4.35 (1.50) | 4.48 (1.44) | 3.86 (1.68) | 0.161 |
| CHOL*, mean (SD), mmol/L | 3.15 (1.75) | 2.87 (2.00) | 3.22 (1.68) | 0.500 |
| HDL-C*, mean (SD), mmol/L | 0.40 (0.25) | 0.29 (0.11) | 0.43 (0.27) | 0.003 |
| LDL-C*, mean (SD), mmol/L | 0.95 (0.68) | 0.48 (0.41) | 1.07 (0.69) | <0.001 |
| H/L ratio*, mean (SD) | 0.71 (1.04) | 1.36 (1.98) | 0.54 (0.50) | 0.008 |
| Statins usage**, n (%) | 0 (0.00) | 0 (0.00) | 0 (0.00) | 0.999 |
| Ventilation free days#, mean (SD), d | 17.31 (9.01) | 14.64 (9.89) | 18.20 (8.62) | 0.150 |
| Renal replacement therapy, n (%) | 13 (18.31) | 6 (40.00) | 7 (12.50) | 0.014 |
| Hospital cost, mean (SD), CHY | 176,782.79 (151,340) | 207,100 (176.901) | 168,662 (144,453) | 0.386 |
| ICU LOS, mean (SD), d | 21.82 (22.08) | 20.60 (20.07) | 22.41 (22.67) | 0.812 |
| Hospital LOS, mean (SD), d | 31.20 (27.08) | 23.87 (21.71) | 33.16 (28.17) | 0.241 |

APACHE: Acute Physiology and Chronic Health Evaluation; CHOL: cholesterol, HDL-C: High-density lipoprotein cholesterol, LDL-C: Low-density lipoprotein cholesterol, H/L: HDL-C/ LDL-C, LOS: length of stay

*on admission

#within 28 days
